# Factors associated with back pain in children aged 6 to 12 years of age, an eight months prospective study

**DOI:** 10.1038/s41598-021-04060-7

**Published:** 2022-01-12

**Authors:** Claire Henriot-Jéhel, Jocelyn Lemire, Caroline Teulier, André Bussières, Arnaud Lardon

**Affiliations:** 1 Université Paris-Saclay CIAMS, 91405 Orsay, France; 2 Université dOrléans CIAMS, 45067 Orléans, France; 3 Institut Franco-Européen de Chiropraxie, 24 Boulevard Paul Vaillant Couturier, 94200 Ivry sur Seine, France; 4 Université du Québec À Trois-Rivières, Trois-Rivières, Canada; 5 Faculty of Medicine and Health Science, McGill University, Quebec, Canada

**Keywords:** Epidemiology, Paediatric research

## Abstract

Associated factors of back pain (BP) development before puberty and its persistence are poorly documented. We investigated the association and possible temporality between prior BP history (PBPH), muscular endurance (ME), aerobic capacity (AC), sport activity variables (SAV) and BP in children aged 6 to 12. We collected baseline characteristics (demographics, PBPH, ME, AC and SAV) of children from three primary schools in Canada. Parents replied to weekly text messages regarding their children BP status over an 8-month period. Logistic regression models were adjusted for potential confounders. Data from 242 children (46% female; 8.6 ± 1.7 years) were included. Over the 8-month survey BP prevalence was 48.1%, while the cumulative incidence was 31.9%. The occurrence of at least one BP event was associated with PBPH [OR (IC 95%) = 6.33 (2.35–17.04)] and high AC [2.89 (1.21–6.90)]. High AC was also associated with the development of a first BP episode [2.78 (1.09–7.07)], but ME and SAV were not. BP appears to be relatively common before puberty. BP history seems to be strongly associated with BP recurrence in children. Aerobic capacity is associated with first BP episode development.

## Introduction

Back pain (BP) is one of the most frequent musculoskeletal disorders in adults and represents a critical public health issue^1^. Indeed, BP can result in significant physical and psychological burden, and high societal cost^2^. Approximately half (49.6%) of the years lived with disability originates from BP alone^3^.

Youth is a turning point regarding BP development. Recent evidence suggests that BP starts early, during childhood and adolescence. A 3-year prospective cohort study of 1465 children aged 8 to 15 years followed weekly using text messaging estimated the BP annual prevalence to approach 30%^4^.

To date however, elements of the causal relationship with BP first occurrence have not been well explored^5^. One crucial (but insufficient) criterion of the 9 Bradford Hill criteria for causality, is temporality. To infer causality, exposure must precede the outcome of interest. Recent literature concerning the causality of low back pain (LBP) does not differentiate between the ‘disease’ of LBP and its recurring episodes mainly due to a lack of a clear definition of absence of LBP at baseline^5^. Thus, reporting whether the child has a prior history of BP at the time of enrolment is necessary.

Furthermore, BP during youth is a predictor of BP in adulthood^6^. Consequently, exploring predictors of BP during childhood and adolescence may help design more effective early interventions to influence or manage its trajectory. Despite the large number of potential factors investigated to date, the causes of early BP onset remain unclear^7^. Linear growth during puberty, physical activity level and physical capacity are some of the main factors examined thus far.

In young people, the puberty period has been highlighted as a critical period for BP occurrence^8,9^. Linear growth during puberty appears to be an independent risk factor for the development of spinal pain, those undergoing greater growth experience increased spinal pain frequency and duration^10^.

Prior studies have also reported physical activity behaviour to be associated with BP in young people. Interestingly, moderate intensity physical activity seems to be associated with a decrease of BP, while more vigorous intensity may increase its incidence^11,12^.

Objective physical capacity factors such as aerobic capacity and muscular endurance are often considered as plausible BP predictors. However, studies have provided inconsistent results. A systematic review^13^ exploring the association between aerobic capacity and BP in children included three longitudinal studies. One reported a positive association, but this association disappeared when the results were adjusted for muscular endurance^14^. Cardon et al.^15^ found a protective association, while a more recent study by Perry et al.^16^ found that the participants with a higher aerobic capacity reported more BP episodes. In addition, a 2007 systematic review^17^ showed no association between trunk muscular endurance and LBP occurrence. In contrast, a more recent meta-analysis^13^ suggested that an efficient trunk extension muscular endurance may be a protective factor for BP in children and adolescents^13^.

This conflicting evidence among available studies may be explained by sample sizes variations, the age range of the study populations (e.g., 9–10 vs. 15–16), confounders adjusted for in the data analysis, or recall bias.

The primary objectives were to estimate the prevalence and the cumulative incidence of back pain in the study population, and to explore the association between BP events and prior history of BP, physical capacity factors including muscular endurance and aerobic capacity and sport activity variables, over an 8-month weekly SMS survey in children aged 6 to 12. We secondarily aimed to determine if these factors possibly are associated with the occurrence of first BP episode.

## Method

### Design

This prospective cohort study gathered baseline data from children attending three different elementary schools in October 2017, and followed them each week over eight months between November 15th 2017 and June 26th 2018. We used the *Strengthening the Reporting of Observational Studies in Epidemiology (STROBE) Statement* to report study results^18^.

### Ethics approval and informed consent to participate

All methods were carried out in accordance with the Declaration of Helsinki ethical principles for research involving human subjects. This study was approved on June 13, 2017 by the Research Ethics Committee for Human Beings at the Université du Québec à Trois-Rivières [CER-17-235-07.06]. Informed consent was obtained from all subjects and/or their legal guardian(s).

### Participants and setting

A convenience sample of 451 children aged 6 to 12 years old attending three elementary schools; two public (*n* = 328) and one private (*n* = 123), were eligible to participate in this study in a town included 137,188 inhabitants at the time of the data collection.

### Eligibility criteria

To be included in the study, the child had to be in good general health as to allow data collection, be between age 6 to 12 years, and one parent had to be reachable via text messaging. Children with physical disabilities or illnesses that contraindicated physical effort were excluded.

### Procedure

School principals were initially contacted by phone by a team member. Subsequently, the nature of the project was fully explained in person. An explanatory brochure of the research project was then provided to all the parents. Those agreeing for their children to participate signed an informed consent form. Even after consenting, children could choose not to participate in one or several physical activities or abandon the study altogether without any consequence.

### Baseline data collection and independent variables

**Figure 1:**
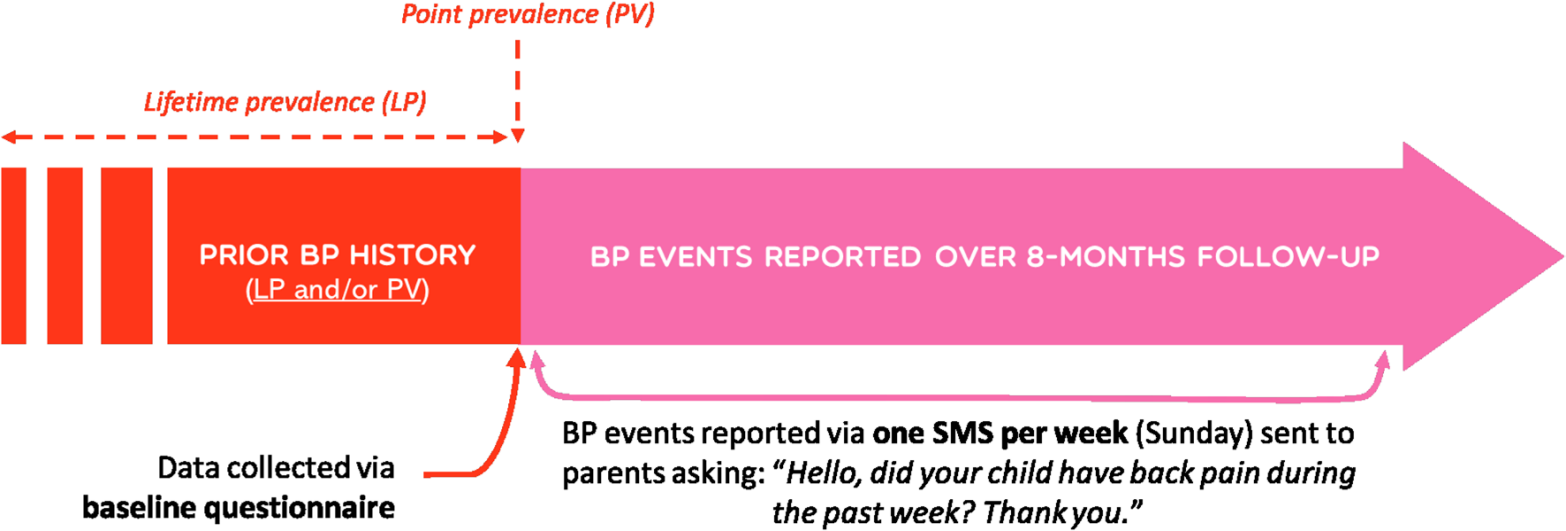
Prior BP history and BP events reporting.

At inclusion, a baseline questionnaire was completed at home by parents to gather contact information (mobile phone number and email address), as well as, the child history of BP and sport activities performed. More specifically:

***Prior BP history*** was assessed to differentiate a first BP episode from any other BP event (i.e. BP reported at any other time point during study). BP history at baseline served to estimate *BP point prevalence (is the child having BP at the time of study enrolment?),* and BP before study onset to estimate the *lifetime prevalence (has the child ever had BP prior to enrolment?)* (Fig. 1).

***Muscular endurance*** was measured by a static test called the abdominal board test, without time limit. This test has been shown to be feasible, valid and reliable for assessing torso muscle endurance for children 8 to 12 years of age (Boyer et al., 2013). It consists of measuring the time during which a participant can maintain the board position. Participants were warned whenever their board position was relaxing. At the second warning, the endurance time (seconds) was noted and the child had completed his test. The test was performed twice and the longest time was retained.

***Aerobic capacities*** were measured using the Andersen test^19^, a reliable shuttle-running test adapted and validated for the age of our sample^20^. Children were asked to run as quickly as possible for 15 s, then to remain still for the next 15 s. They were asked to repeat this sequence (15 s run, 15 s still) during a 10-min period. The results of this test were reported in total length (meter) covered for each child. The greater the distance the child covered in 10 min, the greater was the aerobic capacity.

***Sport activity variables*** such as *sport activity performance* (yes/no), and if yes, the *number of sports practiced* and the *frequency per week* were collected. Only sport activities happening outside of school’s regular physical education classes (e.g. hockey, soccer or swimming) were taken into consideration.

With the participation of physical education teachers who helped clarify instructions to the children, other exploratory variables listed below were collected by experienced chiropractors and graduate students at the three different schools during the children’s physical education classes. A manual explaining each physical test to be carried out by children during these sessions was given to physical education teachers beforehand. All measurements were performed systematically in the same order.

### Confounding factors

Sex, age and a growth spurt occurrence during the course of the study have been identified as possible confounding factors^7,10,21^. A growth spurt was defined as the presence of a whole-year peak height velocity of at least 9 cm/y in girls and 10 cm/y in boys^22^. To determine whether a “growth spurt” occurred during the course of this 8-month study, a proportional peak height velocity had to reach 6 cm/8 months in girls and 6.7 cm/8 months in boys. Anthropometric measurements including height (cm) and weight (kg) were measured twice using a tape measure and a digital scale respectively, once at baseline and again after eight months.

### Back pain outcomes: prevalence and cumulative incidence (dependent variables)

**Figure 2:**
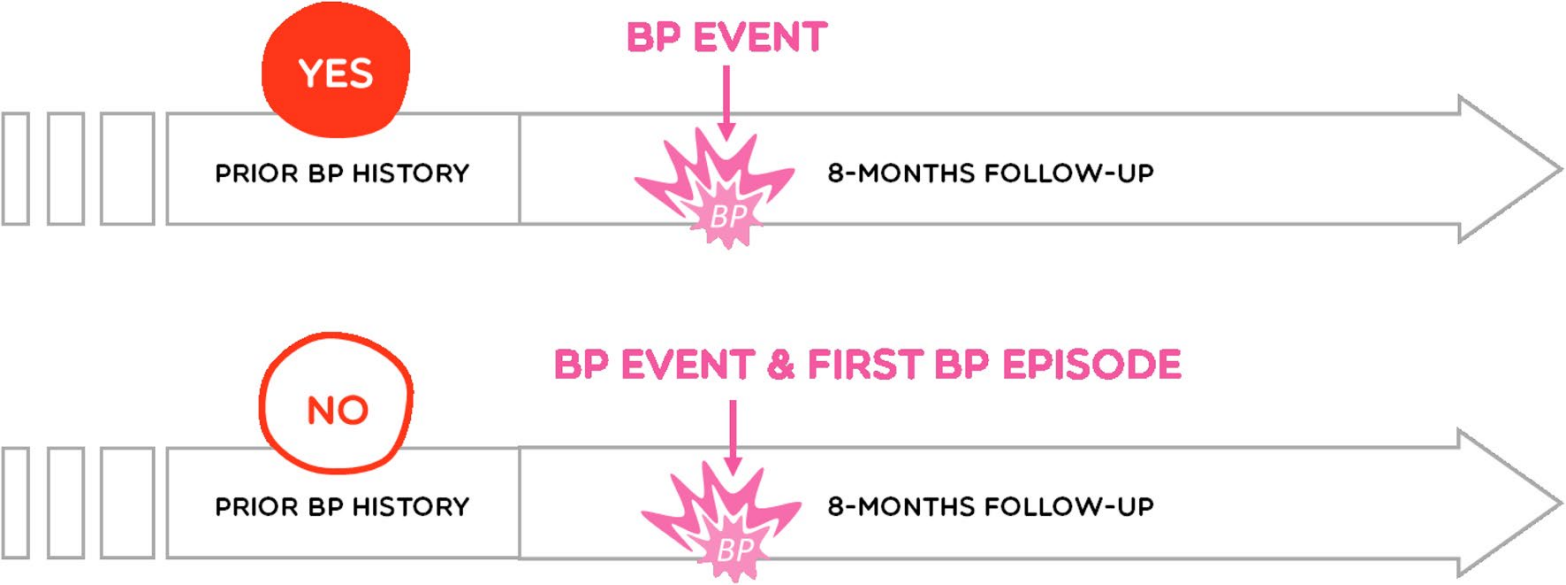
BP event and first BP episode.

In the current study, ***prevalence*** was defined as number of responders reporting the occurrence of at least one event of BP during the 8-month follow-up over the total number of responders (Fig. 1). **BP event** was defined as pain felt in the back at any time point during 8-month follow-up for both children with or without prior back pain history. A diagram of the three regions of the spine (neck/cervical, mid-back/thoracic, lower back/lumbar) was given to the parents to indicate accurately the painful regions. BP events were collected weekly using the SMS Factor platform, a web-based text messaging system, over an 8-month period. SMS systems allow for a more precise estimate of the incidence of BP, having a high response rate and limiting the recall bias (Dissing et al., 2017). Each week parents received the following text message: “*Hello, did your child have back pain during the past week? Thank you.*” Parents had to choose one or more of the proposed answers: (N = no spine pain, 1 = neck pain, 2 = mid back pain and / or 3 = low back pain). In case of non-response, a reminder was automatically sent 48 h later. Traumatic causes of BP event were not specified during the collection of BP data (Fig. 2).

In contrast, ***cumulative incidence*** was defined as the proportion of children (without back pain history at inclusion) who experienced a first BP episode during the 8-month follow-up. Here, a **first back pain episode** was considered when a child without any prior back pain history at baseline reported a first back pain episode during the 8-month follow-up (Fig. 2).

### Statistical analysis

All analyses were performed by a statistician using Stata 15.1. Chi-square tests of independence compared the study variables of follow-up responders (i.e., participants whose response rate to the 32 weeks SMS survey reached at least 70%) to those who were lost during follow-up (i.e., participants who did not reach a 70% response rate over 32 weeks and were excluded from the follow-up analyses).

Prior BP history was reported as a binary variable (present or absent history of BP at enrolment or prior to enrolment). BP outcomes were reported and synthetized as presence or absence of at least one BP event during the 8-month tracking. Aerobic capacity and muscular endurance data were categorized as follow: low (inferior to the first quartile), medium (between the first and the third quartiles) or high (superior to the third quartile). Sport activity performance was reported as a binary variable (yes/no). Both the number of sports practiced and weekly sports activities or training sessions were classified in three categories (none, 1–2 or more than 3). Lastly, the occurrence of a “growth spurt” was reported as a 'yes/no' variable.

Tests for bivariate correlation were performed to determined associations between the potential associated factors and the presence of a BP event during the 8-month tracking period. Independent variables with a degree of statistical significance inferior to 0.2 were included in the stepwise multivariate regression model, and adjusted for three confounding factors: sex, age and occurrence of a “growth spurt”. Non-significant variables (*P* > 0.05) were removed from the model starting with the one with the lowest association level to end up with a model comprising significant variables only. Similar analyses were performed on participants without prior BP history to determine temporality associated factors with a first BP episode and temporality. The stepwise multivariate regression model allowed us to calculate odds ratios. The level of significance was *p* < 0.05 for all analyses unless otherwise specified.

## Results

### Baseline characteristics

Of the 451 children aged 6 to 12 years invited to participate in the study, 242 (53.7%) returned a signed consent form and the completed baseline questionnaire. Of those, 131 were boys (54%) and 111 were girls (46%) with a mean age of 8.6 ± 1.7 [range 6.0–12.0] years. The mean weight was 30.7 (± 8.1) kg [range 17.0–63.0] and the mean height was 135.5 (± 11.7) cm [range 110.0–166.0] with a mean body mass index (BMI) of 16.5 (± 2.3) kg.m^2^ [range 12.1–25.8].

The mean aerobic capacity was 726.0 ± 161.1 m [range 80.0–860.0] for the low category, 943.7 ± 45.4 m [range 868.0–1036.0] for the medium category and 1100.2 ± 62.0 m [range 1040.0–1418.0] for the high category. The mean muscular endurance was 46.0 ± 14.2 s [range 5.0–63.0] for the low category, 91.5 ± 19.7 s [range 64.0–136.0] for the medium category and 218.2 ± 80.0 s [range 137.0–603.0] for the high category. Table 1 displays the sample baseline characteristics.

**Table 1:** Baseline characteristics of included participants and participants who responded to at least 70% of the SMS survey.

	All participants	Follow-up responders *(*> *70% of SMS survey)*
Sample, *n* (%)	242	160 (66.5)
Gender, *n* girls (%)	111 (46%)	77 (48.4)
Age, mean ± SD [range], years	8.6 ± 1.7 [6.0–12.0]	8.6 ± 1.7 [6.0–12.0]
Weight, mean ± SD [range], kg	30.7 ± 8.1 [17.0–63.0]	30.7 ± 8.4 [18.0–63.0]
Height, mean ± SD [range], cm	135.5 ± 11.7 [110.0–166.0]	135.3 ± 11.9 [110.0–166.0]
BMI, mean ± SD [range], cm	16.5 ± 2.3 [12.1–25.8]	16.4 ± 2.3 [12.1–25.2]
Practice of a sport activity, *n* (%)	194 (81.5)	137 (86.2) *
Number of sports practiced, *n* (%)		
None	45 (18.9)	22 (13.8)
1–2	131 (55)	89 (56.0)
≥ 3	62 (26)	48 (30.2) *
Sport practice frequency/week, *n* (%)		
None	45 (19.2)	22 (14.0)
1–2	91 (38.7)	64 (40.8)
≥ 3	99 (42.1)	71 (45.2) *
BP lifetime prevalence, *n* (%)	46 (19.7)	29 (18.5)
BP point prevalence at baseline, *n* (%)	20 (8.6)	14 (8.9)
Prior BP history, *n* (%)	51 (21.8)	32 (19,9)
Aerobic capacity, mean ± SD [range], meter		
Low category	726.0 ± 161.1 [80–860]	743.4 ± 150.7 [80–860]
Medium category	943.7 ± 45.4 [868–1036]	941.0 ± 45.2 [868–1036]
High category	1100.2 ± 62.0 [1040–1418]	1103.7 ± 70.0 [1040–1418]
Muscular endurance, mean ± SD [range], sec		
Low category	46.0 ± 14.2 [5–63]	46.0 ± 12.6 [7–63]
Medium category	91.5 ± 19.7 [64–136]	89.3 ± 19.7 [64–136]
High category	218.2 ± 80.0 [137–603]	212.5 ± 92.2 [137–603]
Growth spurt, *n* (%)	43 (20.6)	30 (21.6)

*Significant difference (*p* < 0.05) between study participants and responders with over 70% of SMS survey data over the course of the study.

At baseline, 19.7% of the 242 children reported BP before study onset (lifetime prevalence). The point prevalence or the proportion of participants who reported BP at study onset was 8.6%. BP *before* and/or *at* study onset (prior BP history) was reported by 51 children (21.8%).

### Follow-up characteristics

The average weekly response rate to the SMS survey was 68.7% over the 32 weeks with a total of 5284 observations recorded altogether.

**Figure 3:**
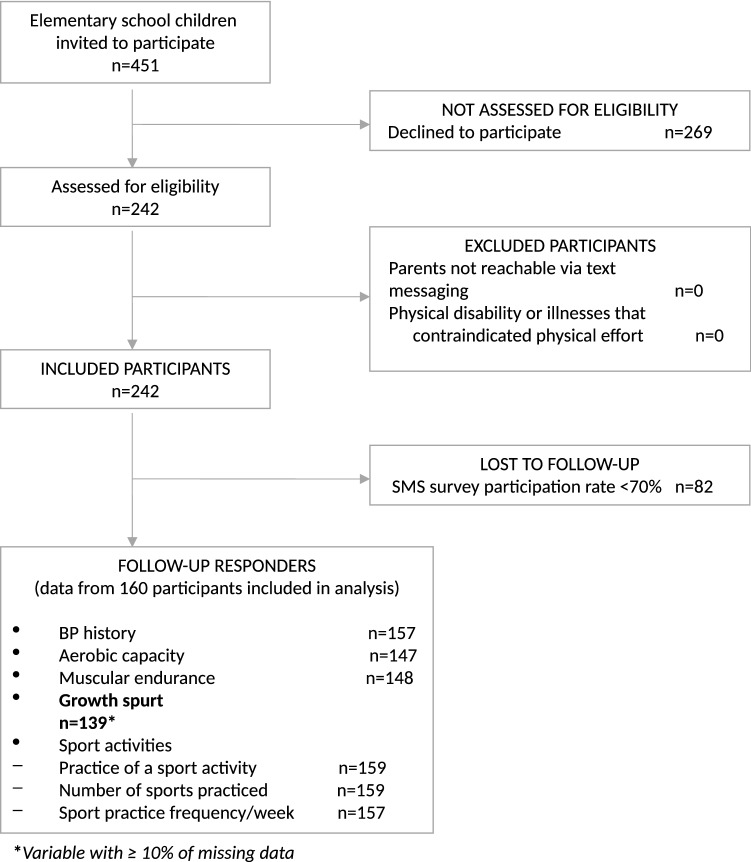
Flowchart of study participants.

**Figure 4:**
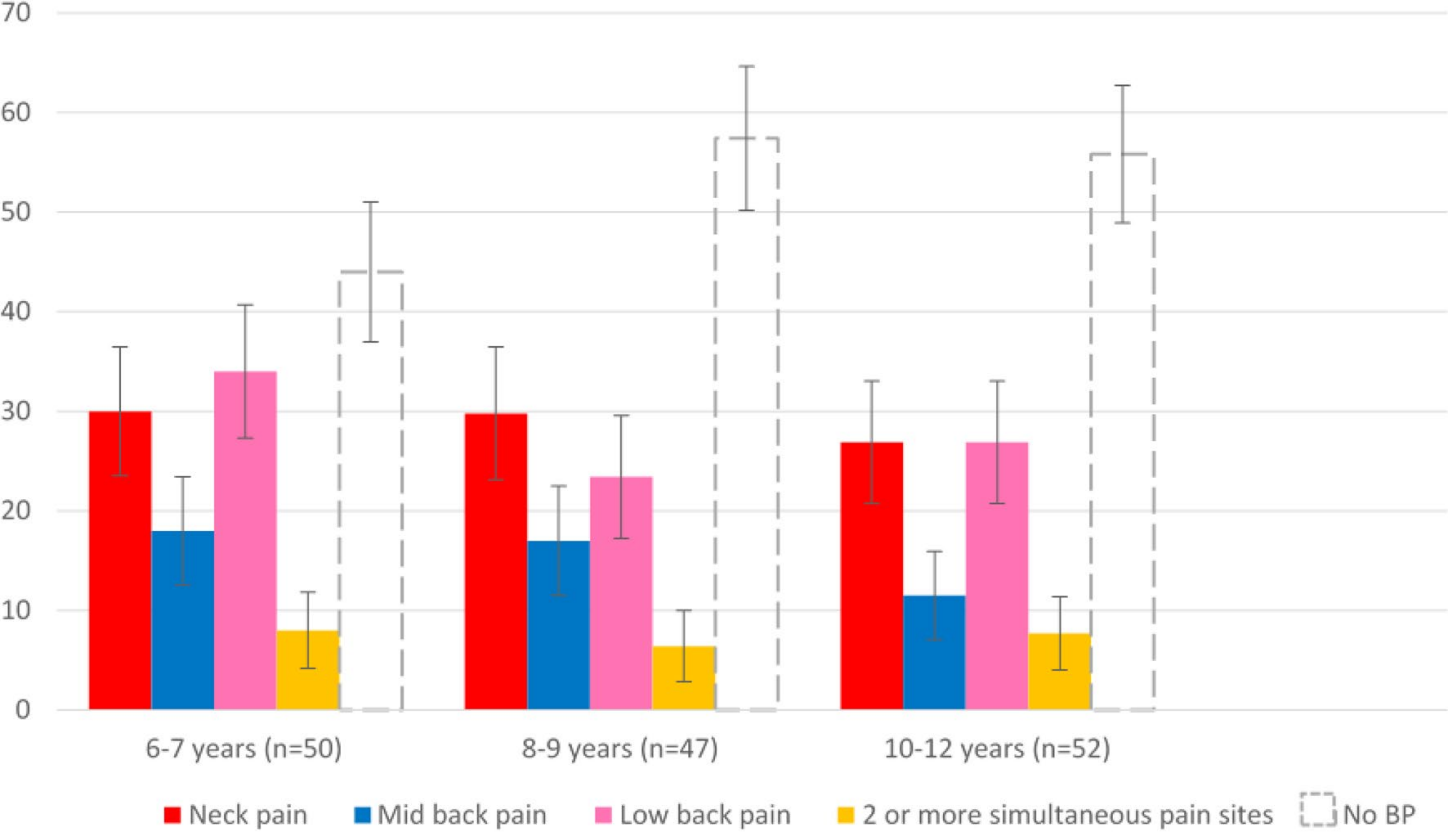
Proportions (standard error) of children with and without BP during the SMS survey as a function of age and pain location(s).

Among the 242 participants, 160 (66.1%) reached a response rate of at least 70% to the 32-weeks SMS survey and were included in the statistical analysis (Fig. 3). This responder group included 83 boys (51.5%) and 77 girls (48.5%) with a mean age of 8.6 ± 1.7 [range 6.0–12.0] years, and was followed an average of 30.9 ± 2.3 [range 23.0–32.0] weeks. Forty-five participants (18.6%) never responded to the SMS survey (Fig. 4).

No significant difference was found between the follow-up responders (i.e., the group with a participation rate > 70%) and those lost to follow-up (*n* = 82) on baseline characteristics, except for sport activities. Compared to participants with a lower participation rate, responders had a higher proportion of children who were involved in sport activities (72.2% vs 86.2%, *p* = 0.009), practiced three or more different sports (17.7% vs 30.2%, *p* = 0.008), and practiced sport at least three times per week (35.9% vs 45.2%, *p* = 0.017) (Table 1).

At eight months after enrolment, the mean weight of the responder group was 32.9 ± 9.1 [range 16.3–67.1] kg and the mean height was 140.4 ± 10.7 [range 115.0–171.0] cm with a mean BMI of 16.5 ± 2.5 [range 9.1–28.1] kg.m^2^. Of those, 21.6% presented a peak height velocity of at least 6 cm/8 months for the girls and 6.7 cm/8 months for the boys, i-e; a “growth spurt”.

Over the 8-month follow-up period, the prevalence was 48.4%, while the cumulative incidence was 31.9%. About half of the children (48.1%) never experienced BP in their life, neither prior to enrolment, nor over the course of the study. Only 6 of the 160 children (3.8%) with a prior history of BP reported having no BP event over the course of the study (Table 2). The BP prevalence among female and male participants did not vary significantly (*p* = 0.927), with a prevalence of 23.1% and 25% respectively.

**Table 2:** BP description during the 8-month follow-up, and proportion of children who never reported any BP among responders (*n* = 159).

BP prevalence during the 8-month follow-up, *n* (%; 95% CI)	77 (48.4; 40.7–56.2)
Never reported any BP before or during the study, *n* (%; 95 CI)	76 (48.1; 40.3–55.9)
Prior history of BP, but none during follow-ups, *n* (%; 95% CI)	6 (3.8; 0.81–6.8)
BP cumulative incidence (first BP episode) at 8-month, *n* (%)	51 (31.9)

The mean number of weeks with back pain was 1.8 ± 2.8 weeks [range 0–16]. Over half of the children (51.9%) in the responder group did not report any BP event during the 8-month follow-up period. The proportion of children reporting BP only once during the survey was 20%, and 13.5% reported BP six times or more (Table 3).

**Table 3:** Proportion of children reporting BP during the study period among responders (*n* = 160).

Number of weeks with BP, mean ± SD [range]	1,8 ± 2,8 [0–16]
Children who never reported any BP during survey, *n* (%; 95% CI)	83 (51.9; 44.1–59.6)
Children who reported BP once, *n* (%; 95% CI)	32 (20; 13.8–26.2)
Children who reported BP 2–5 times BP, *n* (%; 95% CI)	24 (15; 9.5–20.5)
Children who reported BP between 6–10 times, *n* (%; 95% CI)	17 (10.6; 5.9–15.4)
Children who reported BP > 10 times, *n* (%; 95% CI)	4 (2,5; 0.8–4.9)

Furthermore, neck pain and low back pain 8-month prevalence were equally predominant at 28.8%, followed by mid-back pain at 15%. Only 7.5% of the participants reported pain in two regions or more simultaneously at least once. The proportion of children with and without BP events during the SMS survey as a function of age and pain location(s) is presented in Fig. 2.

### Back pain associated factors

Table 4 lists the results of the logistics regression model predicting at least one BP episode during the follow-up period. Table 5 lists the results of the logistics regression model predicting a first BP episode during the follow-up period.

**Table 4:** Association between Prior BP history, muscular endurance, aerobic capacity, sport activity variables and presence of at least one BP event during the SMS survey (*n* = 160).

Independent variables		Odds ratio	95% IC	*P* value
Prior BP history^a^	No	1		
	Yes	6.33	[2.35–17.04]	< 0.000 *
Muscular endurance (s)^b^	-	-	-	-
Aerobic capacity (m)^a^	Low	2.09	[0.89–4.92]	0.091
	Medium	1		
	High	2.89	[1.21–6.90]	0.017*
Sport activity^a^	No	1		
	Yes	1.87	[0.54–6.47]	0.323
Number of sports practiced^a^	None	1		
	1–2	1.26	[0.46–3.5]	0.65
	≥ 3	1 (omitted)		
Frequency of sport activities/week^a^	None	1		
	1–2	0.49	[0.18–1.36]	0.172
	≥ 3	1 (omitted)		

* *p* < 0.05; ^**a**^ Variables with *p* < 0.2 to the bivariate regression model were added to the multivariate regression model and adjusted for age, sex and growth spurt occurrence; ^b^: variables with *p* > 0.2 in the bivariate correlation test and thus not included in the stepwise multivariate regression model.

**Table 5:** Association between muscular endurance, aerobic capacity, sport activity variables and first BP episode incidence during the SMS survey (i.e., only follow-up responders without BP at study onset, *n* = 128).

Independent variables		Odds ratio	95% IC	*P* Value
Muscular endurance (s)^b^	-	-	-	-
Aerobic capacity (m)^a^	Low	1.95	[078–4.94]	0.154
	Medium	1		
	High	2.78	[1.09–7.07]	0.032 *
Sport activity^b^	-	-	-	-
Number of sports practiced^b^	-	-	-	-
Frequency of sport activities/week^a^	None	1		
	1–2	1.38	[0.37–5.07]	0.630
	≥ 3	2.22	[0.63–7.81]	0.213

* *p* < 0.05; ^**a**^ Variables with *p* < 0.2 to the bivariate regression model were added to the multivariate regression model and adjusted for age and sex; ^b^: variables with *p* > 0.2 in the bivariate correlation test and thus not included in the stepwise multivariate regression model.

### Prior BP history

#### Association with BP event over eight months

Prior BP history was associated with the occurrence of at least one BP event during the 32 weeks SMS survey [OR (95% CI) = 6.33 (2.35–17.04), *p* = 0.000] (Table 4).

### Aerobic capacity

#### Association with BP event over eight months

High aerobic capacity was associated with the occurrence of at least one BP event during the 32 weeks SMS survey [OR (95% CI = 2.89 (1.21–6.90), *p* = 0.017] after adjusting for age, sex and growth spurt occurrence. The categories defined as low and medium did not indicate a significant association with BP outcomes (Table 4).

#### Association with a first BP episode during the SMS survey

High aerobic capacity was also associated with the development of a first episode of BP during the 32 weeks SMS survey [OR (95% CI = 2.78 (1.09–7.07), *p* = 0.032]. The categories defined as low and medium did not indicate a significant association with BP outcomes (Table 5).

### Muscular endurance

Muscular endurance showed no significant association with either the occurrence of at least one BP event, nor the development of a first BP episode during follow-up (Tables 4 and 5).

### Sport activity variables

Sport activity performance, the number of sports practiced and practice frequency per week showed no significant association with either the occurrence of at least one BP event reports, nor the development of a first BP episode during follow-up (Tables 4 and 5).

## Discussion

At baseline, nearly 20% of the children reported a prior episode of BP and 8.6% admitted experiencing BP among the 242 participants. In contrast, nearly half of the 160 children (51.1%) reported at least one BP event over the 32 weeks follow-up, and the study cumulative incidence was 31.9%. NP and LBP were the most frequent pain locations, while MDP was less common. Over half of the children never reported any BP (either before or during the study). Interestingly, a prior history of BP and a high aerobic capacity are associated with at least one BP event reported during the 32 weeks SMS survey, while a high aerobic capacity was associated with a first BP episode occurrence.

### Prevalence

While historically considered a rare and potentially serious condition, research over the past decades has shown that back pain in children is a common disorder^23^. For instance, a Danish National Birth Cohort study of 46,726 11–14-year-olds reported severe spinal pain (neck, middle back, and low back pain) in 9.8% of boys and 14.0% of girls, and moderate spinal pain in approximately 30% of all children^24^. Intriguingly, a 2007 review of BP lifetime prevalence reported estimates ranging from 5 to 74% in studies of adolescent^25^. Such a wide variation is partly due to: how back-pain is defined, differing methods of data collection and lengths of recall period. For these reasons, it seems difficult to compare our results with other published studies. However, a previous prospective cohort study using similar BP data collection method (SMS track) and recall period on a children population over a 2.5-year period^26^ reported that three quarters of the children never experienced any back pain. In contrast, our study found that about half of participants never experienced any BP. Such difference might be due to the fact that the former prospective cohort study asked both for spinal, upper limb and lower limb pain, while the present study focused solely on spinal pain.

### BP history

Our findings suggest that children with a history of BP are six times more likely to report BP at follow-up. Similarly, the review by Beynon et al.^27^. reported that a having a history of back pain predicts further back pain in late adolescence and in adulthood (odds ratios ≥ 2.7). Participants included in these longitudinal studies were older (≥ 12 years) than our population^6,28,29^. Despite the short follow-up period and small sample, our findings suggest that BP in young children may also be associated with BP event in early adolescence.

### Aerobic capacity

In the current study, children with a high aerobic capacity, measured using the Andersen test^19^, had greater than a two-fold increase risk of BP (cumulative prevalence) and of having a new episode of BP (cumulative incidence). Interestingly, a review by Lardon et al*.*^13^ found conflicting results from three cross-sectional studies, showing no association^14^, a negative association^15^ and a positive association^16^.

### Muscular endurance

Muscle endurance measured in our sample using the abdominal board test, without time limit did not show any association with BP. In contrast, a review and meta-analysis by Lardon et al*.* 2015 reported a protective association between back muscle endurance in extension, and back pain (OR = 0.75, 95% CI 0.58–0.98)^13^. All included studies in this review assessed back muscle endurance in extension using the Biering-Sorensen test^30^ while in the present study we used the abdominal board test following instructions from a Canadian guide for children physical skills assessment^31^.

### Physical activity

Using self-reported measures of physical activity level, our study did not show any association between the type and number of sport activities or the frequency of activities per week and the occurrence of BP. Similarly, Aartum et al.^32^ school-based longitudinal cohort study found neither cross-sectional (*n* = 906) nor longitudinal (*n* = 625) associations between physical activity, measured using accelerometers, and spinal pain (neck pain, mid back pain, LBP) in 11–15 year old Danes adolescents. In contrast, a prospective cohort of 1160 school age Danish children (age 6 to 12 years) found that greater physical activity was associated with increased occurrence of spinal pain. However, the nature of this relationship depended on the intensity of activity. Increased time in vigorous physical activity predicted future spinal pain, while increased time in moderate intensity activities tended to protect against spinal pain^12^.

A recent scoping review of potential risk factors or triggers *for BP (first time, recurrent, and ongoing BP)* in children and young adults identified the female gender, older age, a history of BP, late pubertal status, a family history of back pain, and increased growth spurt, as likely risk factors. There is limited research on muscle endurance in the development of BP^27^. Potential risk factors or triggers for *incident and episodic BP* included female gender and older age, as well as increased / vigorous intensity physical activity and psychosocial factors. No association or weak associations were found for body mass index, height, muscle strength, smoking, and systemic/illness factors^27^.

### Strengths and limitations

Strengths of this study are a prospective design and the availability of information on several factors studied within the same study sample. The cohort design of the study enabled us to include data from questionnaires at different point in time, including the assessment of prior history of spinal pain.

Nonetheless, this study has several limitations. First, the main outcome, spinal pain, was studied based on a parent reported questionnaire which may affect the reliability of the data. It can be challenging for children to describe the presence and type of pain and for the parents to systematically report children’s complaints. However, parents decide whether or not to restrict or motivate their children to engage in their activities, based on their interpretation of their child’s pain^33^. In our study the risk of excessive pain reporting seems unlikely, nonetheless, perhaps more pain episodes might have been reported if the children had responded themselves^34^. Furthermore, despite an effort to standardize the definition of an episode of BP to the participants and their parents, the lack of specification of the traumatic or incidental nature of the BP notified by the participants induces a risk of measure bias for this variable. Also, the results on pain location might not strictly represent spinal pain. Although a pain diagram was included in the questionnaire, the source of pain might be in some cases non-MSK pain.

The sport activity variable’s collection might have suffered from an unclear wording of the questions encouraging a subjective interpretation, especially regarding the definition of the activity’s intensity.

Finally, a large number of participants were lost during follow-up according to our responder definition of over 70% response rate across the 32 weeks SMS. The collection of mobile phone numbers on paper format implied a risk of error both in the decryption of some handwriting, as well as, in the manual transcription of the numbers. Since the potential for human error is not negligible, it could explain part of the 18.6% of participants who never answered the survey along with potential technical concerns with mobile phones or telephone plans and participation retraction. Indeed, some parents may have deemed unimportant to respond if their child felt no pain, resulting in an overestimation of the prevalence. Because the missing values in the multivariable analysis were selective (i.e., not random), imputation was not suitable. Furthermore, no inferences can be made on causality or direction of associations due to the cross-sectional approach of the analyses. As selection bias towards a healthier population and higher socio-economic status might have occurred, results should be interpreted with caution.

Other methodological considerations include the following: we could not contact parents directly, likely influencing recruitment and retention rates. In addition, intake questionnaire instructions regarding types and frequency of sport activities were unclear as some parents reported on active transportation (e.g., cycling to school) or did not report total time of weekly sports practiced per week for all sports they listed (e.g., soccer but not for hockey). While muscular endurance was measured at the school, encouragements received from team members in small groups may have varied.

### Future research

Future studies should document both the presence and the intensity level of back pain reported by the children using validated pain measuring tools^35^, and determine a pain threshold (e.g., ≥ 2/10 on the VAS) for inclusion at any point in time of the study. Our finding suggests that 21.8% of participants already experienced prior BP at baseline. Future studies should seek to include preschoolers and follow them throughout the lifespan to determine BP trajectory considering potential influence of socioeconomic and psychosocial factors. For instance, a recent Danish study found that children living in less-educated or lower-income families, not living with both of their parents, and those without biological full siblings were more likely to experience spinal pain^24^. Recent reviews also suggest that adverse childhood experiences (ACEs) such as child maltreatment and exposure to domestic violence are associated to long-term causes of ill health, including depression, cancers, cardiovascular disease, and diabetes, and possibly chronic spinal pain and disability in later life^36–38^. High quality longitudinal studies should seek to determine the effect of unique and cumulative ACEs (number and type) as risk factors for persistent pain and disability in adolescence and adulthood.

## Data Availability

The data used and/or analyses during the current study are available from the corresponding author on reasonable request.
